# Dietary Diversity as a Risk Factor for Obesity in Algerian Patients with Type 2 Diabetes Mellitus

**DOI:** 10.3390/healthcare9091229

**Published:** 2021-09-17

**Authors:** Abdenour Bounihi, Hamza Saidi, Asma Bouazza, Hassiba Benbaibeche, Malha Azzouz, Elhadj Ahmed Koceir

**Affiliations:** 1 Laboratory of Alimentation, Transformation, Contrôle et Valorisation des Agro-ressources, Ecole Supérieure des Sciences de l’Aliment et des Industries Agroalimentaires (ESSAIA), Algiers 16004, Algeria; 2 Bioenergetics and Intermediary Metabolism Team, Laboratory of Biology and Physiology of Organisms, Faculty of Biological Sciences, University of Sciences and Technology HOUARI BOUMEDIENE (USTHB), Algiers 16111, Algeria; hsaidi@usthb.dz (H.S.); abouazza@usthb.dz (A.B.); h.benbaibeche@univ-alger.dz (H.B.); ekoceir@usthb.dz (E.A.K.); 3 Department of Nature and Life Sciences, Faculty of Sciences, University of Algiers, Algiers 16000, Algeria; 4 Department of Medicine, Faculty of Medicine, University of Algiers, Algiers 16000, Algeria; m.azzouz@univ-alger.dz; 5 EPH Bologhine Ibn Ziri, Algiers 16090, Algeria

**Keywords:** diabesity, type 2 diabetes, obesity, dietary diversity, Algeria

## Abstract

Although the incidence of “diabesity” (coexistence of type 2 diabetes and obesity) is alarmingly increasing in Algeria, the diet–diabesity link has not been well defined. This study aimed to explore the association between dietary diversity score (DDS) and obesity among Algerian type 2 diabetic patients. It was a cross-sectional observational study involving 390 type 2 diabetic patients. Anthropometric data were gathered, and dietary intake information was obtained through a 24-h dietary recall method, which was used to calculate DDS. Potential confounders such as age, sex, smoking, physical activity and energy intake were controlled for using multivariate logistic regression. A total of 160 patients (41.3%) were classified as obese. As expected, obese patients had a higher body mass index, waist circumference, hip circumference, body fat and fat mass index. Furthermore, obese patients more frequently met carbohydrate recommendations and had a higher intake of meat and protein. Female sex, hypertension, low physical activity and high meat and protein intake were positively associated with diabesity. Additionally, higher DDS was positively associated with diabesity after adjusting for confounders. Thus, a more diversified diet may be a risk factor for obesity among Algerian type 2 diabetic patients.

## 1. Introduction

Diabesity, defined as the coexistence of diabetes mellitus type 2 (T2DM) and obesity, was considered the pandemic of the 21st century up to March 2020 when the World Health Organization (WHO) announced coronavirus disease 2019 (COVID-19) as a pandemic [1]. Epidemiological data show that the number of “diabesity” cases is increasing rapidly in Algeria, where a large proportion (31.51–40.82%) of patients with T2DM are affected by obesity [2,3,4].

The incidence and progression of T2DM are closely associated with body mass index (BMI) [5]. In fact, it is well established that diabetic patients with higher BMI are at increased risk for complications, thrombogenic events, poor response to antihyperglycemic medication and the development and severity of COVID-19 [1,5]. In addition, poor control of cholesterol, blood pressure and blood glucose levels have been reported in such patients [5]. Hence, effective interventions are urgently required to prevent diabesity, and dietary quality has become a focus for these interventions. The dietary diversity score (DDS) is one of the leading dietary indices for evaluating overall diet quality; nevertheless, the relationship between DDS and obesity in the general population remains controversial. Although higher DDS is related to greater intake of healthy food groups [6,7], which are recognized to protect against obesity, consuming a diet with a high variety increases energy intake and the risk of obesity [8,9,10]. However, such significant relationships were not observed in other studies [11,12].

Since most of the above-described studies have been conducted in general population samples, questions remain regarding the link between dietary patterns and obesity in type 2 diabetic patients, especially in the Algerian population who is experiencing accelerating industrialization, urbanization and a nutrition transition concomitant with a dramatic rise in the incidence of diabetes [13]. Thus, assessing the diet quality of obese and non-obese Algerian T2DM patients makes an important contribution to the early identification of dietary patterns associated with increased risk of diabesity and provides evidence for healthcare professionals and policy-makers to plan specific interventions. In this line, the present study was conducted with the aim to assess the relationship between diet quality, as evaluated by DDS and obesity in Algerian patients with T2DM. 

## 2. Materials and Methods

### 2.1. Study Design and Data Collection

This was a cross-sectional observational study conducted among T2DM patients on follow-up at the diabetic clinic “Maison du Diabétique” of Mustapha Pacha University Hospital in Algiers (Algeria), from January to March 2019. A single population proportion formula with an assumption of 95% confidence level, a 5% degree of precision, 40.82% previous prevalence of obesity among T2DM patients from Algeria [4] and a non-acceptance rate of 5% was used to determine a final sample size of 390. Having type 2 diabetes, being 18 years and older and visiting the clinic during the study period were the inclusion criteria. Patients who were pregnant or lactating, who were on a special diet, who were taking medications that could affect appetite or weight, and who reported implausible energy intakes (less than 800 Kcal/d and more than 4000 Kcal/d) [14] were excluded. A systematic random sampling technique was employed to select the study sample. All participants provided written consent for participation in the study, which was conducted in accordance with the ethical guidelines of the Declaration of Helsinki. Ethical review and approval were waived for this study, due to its observational and anonymized nature. Participants were interviewed using a standardized questionnaire to elicit information about demographic, clinical, and lifestyle characteristics. Participants’ body weight, height, waist circumference and hip circumference were measured using a standardized protocol. BMI was calculated as weight in kilograms divided by height in meters squared (kg/m^2^) and waist-to-hip ratio as waist circumference in cm divided by hip circumference in cm. The WHO cut-offs were used to classify participants as normal weight, overweight, obese or abdominally obese [15,16]. Percent body fat, fat mass and fat mass index were also calculated for each participant according to methods described previously [17,18]. Physical activity level was estimated using the International Physical Activity Questionnaire-short version and categorized as low, moderate or high. A detailed medical history was taken by a trained physician and common diabetes-related complications and comorbidities were assessed. The diagnosis of diabetic retinopathy was established by fundus photography. Diabetic nephropathy was diagnosed by the presence of albuminuria and/or low estimated glomerular filtration rate in the absence of signs or symptoms of other primary causes of kidney damage. Diabetic neuropathy was diagnosed if patients fulfilled two or more of the following four criteria: The presence of signs of neuropathy, the absence of ankle tendon reflexes, abnormal scores for pressure and/or vibration perception. Cardiovascular disease was considered in patients who had a history of ischemic heart disease, cerebrovascular disease or peripheral vascular disease. Hypertension was defined as blood pressure ≥140/90 mmHg (average of two readings taken five minutes apart) and/or the use of antihypertensive drugs. Glycemic status was checked by the glycated hemoglobin (HbA1c) level, and it was considered controlled when HbA1c was <7% [19].

### 2.2. Dietary Assessment

Dietary assessment was carried out using a 24-h recall method. Although a single 24-h recall might not accurately reflect the usual intake, this method is considered the best approach for determining dietary diversity, since multiple 24-h dietary recalls result in a lack of accuracy [8]. However, when patients reported that their dietary intake on the previous day was atypical, they selected another day for the interview. All participants provided a detailed record of what they ate and drank during the past 24 h; the amounts consumed were estimated using photographs of household utensils and food portions. For mixed dishes, food groups were determined on the basis of their ingredients. A computer program, NutriSurvey (EBISpro, Willstätt, Germany), was used to calculate energy and macronutrient intake. Then, energy intake was compared with the calculated energy requirement using predictive equations of the Institute of Medicine [20]. Moreover, macronutrient intake (expressed as a percentage of energy intake) was compared with the dietary recommendations for diabetes given by the Diabetes and Nutrition Study Group of the EASD [21].

DDS was used to calculate dietary diversity by applying the procedures described by Kant et al. [22]. Five food groups were considered, namely grains, meats, fruits, vegetables and dairy products. Scores were calculated based on the 24-h recall data by counting one point for each food group consumed by the participant. As recommended by Kant et al. [22], we excluded food groups consumed in amounts below the minimum requirements. Thus, for meat, fruit and vegetable groups, the minimum considered was 30 g for all solid foods with a single ingredient and 60 g for all liquids and mixed dishes; however, for the dairy and grain groups, the minimum considered was 15 g for all solids and 30 g for all liquids and mixed dishes [22].

### 2.3. Statistical Analyses

All data were verified for normality distribution using the Kolmogorov–Smirnov test. Independent Student *t*-tests, Chi-square and Mann–Whitney U tests were used to assess differences between the two groups. The association between diabesity and demographic, clinical, anthropometric and physical activity variables, as well as DDS and food groups intake were evaluated using univariate and multivariate logistic regression. *p* < 0.05 was considered as significant. All statistical analyses were conducted using SPSS, version 25 (SPSS, Inc., Chicago, IL, USA).

## 3. Results

Three hundred and ninety patients with T2DM were enrolled in this study. Their basic characteristics are shown in Table 1. Females were in the majority (71.5%). The mean age was 57.1 ± 10.4 (range 29–88) years. Fewer than half of the participants (40.8%) were 60 years of age and older. Overall, smoking prevalence was only 2.8%. A family history of diabetes was reported by 71.8% of the enrolled patients. Patients were diagnosed with T2DM for a mean of 8.8 ± 7.3 years. Over half of the patients (61%) had had their diabetes for less than 10 years. Of our patients, 2.8% were on a diet alone, 60.8% were on oral antidiabetic agents, 12.1% were on insulin alone and 24.4% were on a combination of oral antidiabetic agents and insulin. The mean HbA1c level was 7.5 ± 1.7%, and 168 (43.1%) patients were below the HbA1c goal (i.e., <7%). The majority of patients (61.1%) had at least one diabetes-related complication/comorbidity and their prevalence rates were as follows: Cardiovascular disease (14.6%), retinopathy (13.3%), neuropathy (2.8%), nephropathy (6.4%) and hypertension (47.2%). According to the BMI criteria, 79 patients (20.3%) were normal body weight, 150 (38.5%) were overweight, 161 (41.3%) were obese and, according to waist circumference values, almost all patients (93.5%) had abdominal obesity. Mean body fat and fat mass index were 40.0 ± 8.8% and 12.1 ± 4.5 kg/m^2^, respectively. With regard to physical activity level, the percentage of patients who were categorized as having low physical activity was 61%, whereas 34.9% and 4.1% were categorized as having moderate and high physical activity, respectively.

Demographic, clinical, anthropometric and physical activity variables associated with diabesity, as identified by univariate analysis, are presented in Table 2. We observed that patients with diabesity were more often female (*p* < 0.0001), hypertensive (*p* = 0.023), less physically active (*p* < 0.0001) and, as expected, their BMI, waist circumference, hip circumference, body fat and fat mass index were higher (*p* < 0.0001) (Table 2).

**Figure 1:**
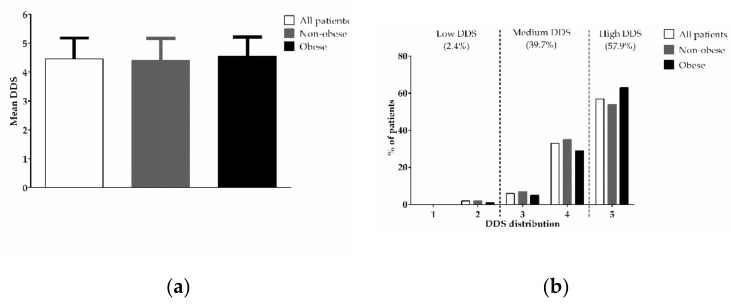
Mean (**a**) and distribution (**b**) of DDS. (**a**) Each bar represents the mean ± SEM; (**b**) each bar represents the percentage of patients with the following DDS: Low (DDS < 3), medium (DDS = 3 or 4) or high (DDS = 5).

The mean and distribution of DDS according to obesity status are shown in Figure 1. The DDS was in the range of 1–5, with a mean value of 4.46 ± 0.72 (Figure 1a). Most participants had high (57.9%) or medium (39.7%) dietary diversity scores (DDS = 5 and DDS = 3 or 4, respectively) (Figure 1b). The mean and distribution of DDS were similar between the two groups (Figure 1a,b).

The dietary intake of the study patients is indicated in Table 3. Food groups consumed by the highest proportion of patients were “grain” (97.7%), “vegetable” (96.7%), “fruit” (92.8%) and “dairy” (89.7%). However, over two-thirds of patients (69.7%) reported consuming foods from the “meat” group. The daily intake of fruit, vegetables, dairy products and grain was similar between the two groups, but obese patients had a higher meat intake than non-obese patients (98.1 ± 87.1 vs. 80.2 ± 78.7g/d, respectively; *p* < 0.05).

The daily energy and macronutrient intakes of the patients are presented in Table 4. Non-obese and obese patients reported energy intakes between 800 and 2806 Kcal/d and 802 and 2358 Kcal/d, respectively. On average, carbohydrates provided 60.6 ± 9.9% of the total energy intake with 43.3% of the patients reaching the recommended levels (45–60%). Compared to non-obese patients, obese patients more frequently met carbohydrate recommendations (49.7% vs. 38.9%, *p* < 0.05). Protein provided, on average, 12.3 ± 5.6% of the total energy intake, and 54.4% of the patients reported a diet with the recommended level of protein intake (10–20%). However, obese patients consumed significantly more protein, expressed as g/d (46.6 ± 22.2 g/d vs. 41.2 ± 22.8 g/d, *p* < 0.05) and as a percentage of total energy intake (13.3 ± 5.6% vs. 11.5 ± 5.5%, *p* < 0.05), than non-obese patients. On average, fat provided 27.1 ± 10.2% of the total energy intake, and 77.2% of the patients met the recommended level of <35% of total energy intake.

Crude and multivariate-adjusted odds ratios (ORs) and 95% confidence intervals (CIs) for the association of DDS and food group intake with diabesity are presented in Table 5. In the crude model, no significant association was found between DDS and diabesity (Table 5). However, after adjustment for age and sex (Model 1), with each unit increase in DDS, the odds of being diabese increased by 37% (OR = 1.376, 95% CI 1.020–1.855; *p* < 0.05). This association remained significant after further adjustment for smoking and physical activity based on Model 1 (Model 2, OR = 1.458, 95% CI 1.073–1.981, *p* < 0.05) and energy intake based on Model 2 (Model 3, OR = 1.426, 95% CI 1.029–1.974, *p* < 0.05). Moreover, in the crude model, higher meat intake was associated with statistically significant increases in the odds of being diabase (OR = 1.003, 95% CI 1.000–1.005; *p* < 0.05). This association was strengthened in Model 1, which was adjusted for age and sex (OR = 1.003, 95% CI 1.001–1.006; *p* < 0.05), after further adjustment for smoking and physical activity based on Model 1 (Model 2, OR = 1.003, 95% CI 1.001–1.006, *p* < 0.05) and total energy intake based on Model 2 (Model 3, OR = 1.003, 95% CI 1.000–1.006, *p* < 0.05).

## 4. Discussion

Diabesity constitutes an important risk factor for the occurrence of short-term and long-term medical complications [23], therefore there is an urgent necessity to institute appropriate measures to control this public health issue. According to the results herein, two out of five diabetic patients were obese. Previous studies reported the high prevalence of obesity in Algerian T2DM patients, with its prevalence ranging from 31.51% in Sidi-Bel-Abbes and Mascara (North-Western Algeria) [2] to 35.8% in Algiers (North-Central Algeria) [3], with the highest prevalence (40.82%) reported in Tebessa (North-Eastern Algeria) [4]. Although genetics contribute significantly to the obesity epidemic, there are other factors that need to be considered, such as the nutritional transition resulting from urbanization and westernization and characterized by unhealthy eating behaviors and sedentary lifestyles [24].

In the present study, female sex, hypertension and physical inactivity were significant predictors of diabesity. These findings are in line with the results of a report made by the Algerian Ministry of Health, Population and Hospital Reform in collaboration with WHO [13], which indicates that obese Algerian women with T2DM lead a significantly more sedentary lifestyle than men (26.5% vs. 17.5%, respectively). In addition, this report indicates that the prevalence of obesity was 33.8% and 10.3% in women and men, respectively, while the prevalence of hypertension was 47.7% and 34.9%, respectively [13]. Potential explanations for these findings include the fact that women are more prone to overweight and obesity owing to physiological events that occur during the reproductive years and that women are less likely to be engaged in activities of moderate or vigorous intensity than men [25].

The current study is the first in Algeria to report that the majority of patients with T2DM had adequate dietary diversity. In addition, while obese and non-obese diabetic patients had similar raw DDS, after adjusting for covariates, the obese group had higher dietary diversity than the non-obese group. According to previous reports [6,7,8,9,10,11,12], DDS is known as a good indicator for assessing the relationship between diet and obesity or diet and health risks. However, to our knowledge, this is the first epidemiologic study that considers the association between DDS and diabesity, thus the results are not easily comparable. Some studies in the general population have reported that DDS is positively related to obesity [8,9,10]. In this regard, a study conducted among Sri Lankan adults revealed that, while DDS increased, in parallel, the percentage consumption was increased in most food groups, and this may have led to increased energy intake and obesity [8]. Comparable findings were observed among Tehran adults [9] and in a sample of Mexican men [10]. In the present study, energy and carbohydrate intake showed a moderate association with DDS, while fat and protein intake showed weak and strong associations, respectively (see Supplementary Table S1). On the other hand, low-glycemic-load foods that are rich in fiber were negatively related to weight gain than high-glycemic-load foods that are poor in fiber; consequently, some other studies have reported a negative relation between DDS and obesity [6,7]. In fact, although these studies found that DDS and energy intake were directly associated, they concluded that the increased energy intake was related to greater consumption of healthy food groups, thus leading to a negative association between DDS and obesity [6,7]. However, some studies have not found any association between DDS and obesity [11,12], probably due, as pointed out by Savy et al. [11], to the fact that higher DDS was related to the consumption of a combination of healthy and unhealthy food products. Several reasons could explain these discordant results, including differences in study populations, the use of different tools for dietary intake assessment, variation in the number of food groups and their subgroups retained for the calculation of DDS and the lack of a standardized scoring method.

This study found that higher meat intake was a significant independent predictor of diabesity, and this is consistent with the literature [23,26]. In fact, in a Mexican study, Easton et al. [26] have shown that obese T2DM patients consumed significantly more meat than non-obese T2DM patients. Another study by Cheung et al. [23] found that obese Chinese T2DM patients had significantly higher meat consumption compared to non-obese T2DM patients. In this line, Duarte et al. [27] investigated the relationship between percentage body fat (PBF) and dietary sources of fat from the usual diet of Brazilian patients with T2DM. These authors found that the consumption of red meat was associated with higher PBF [27]. Furthermore, in the adjusted analysis, these same authors found that the higher tertile of processed meat intake was associated with increased PBF compared to the lower tertile [27]. On the other hand, various genetic factors could be involved in the susceptibility to obesity by affecting an individual’s energy consumption and food preferences. For example, in 2075 overweight or obese participants with T2DM from the Look Action for Health in Diabetes trial, it was found that genetic polymorphisms of the brain-derived neurotrophic factor were associated with greater total caloric intake and more servings from the dairy product and the meat, eggs, nuts and beans food groups [28].

Our results regarding energy intake differ from those of Diaf et al. [29] who reported that overweight/obese Algerian patients with T2DM had an average energy intake of 2212.84 ± 233.59 Kcal/d, which is much higher than we found. However, despite the high prevalence of overweight and obesity in our sample, energy requirements were not met in the majority of patients (see Supplementary Figure S1). Furthermore, our results differ from those of Diaf et al. [29] regarding the average intake of carbohydrates, protein and fat, as these were higher than those found in the present study, especially the intake of protein and fat (89.48 g and 111.43 g, respectively). On the other hand, our results concerning the relative contribution of macronutrients to energy intake agree, to some extent, with those of Diaf et al. [29] who found that carbohydrate, protein and fat contributed 50.07%, 22.2% and 27.6% to total energy intake, respectively. These discordances could be attributed to methodological and/or clinical variations.

Finally, a set of limitations warrant acknowledgement. First, the cross-sectional design of the study establishes associations and not causality. Second, we did not consider biomarkers in our analysis. Third, it was practically difficult to estimate diversity within food groups because the Algerian diet is largely comprised of mixed dishes. Fourth, due to limited data on the micronutrient content of mixed dishes, we were unable to calculate the nutrient adequacy ratio and the mean adequacy ratio. Fifth, educational and economic status were not recorded, although differences in either status are negligible because most participants were retired (similar economic conditions) and had limited access to higher levels of formal education as they were born in the 1940s–1960s. Acknowledging these limitations, our study, to our knowledge, is the first to consider the relationship between DDS and diabesity. The enrollment conditions were well-defined, and various potential confounding factors were considered. The sample was homogeneous in the sense that all participants attended the same clinic and thus had received diabetes education from the same team. In addition, to reduce the effect of recall bias, standardized methods were used. 

## 5. Conclusions

In conclusion, the study shows that, overall, Algerian type 2 diabetic patients had adequate dietary diversity. When adjusting for confounding variables, obese T2D patients had significantly higher dietary diversity than their non-obese counterparts. As we found a medium association between DDS and energy intake, the consumption of a diversified diet should consider the controlling of total energy intake to combat weight gain and diabesity. Moreover, female sex, hypertension, low physical activity and high meat and protein intake were the factors that were found to be associated with diabesity. These findings are important for healthcare professionals and policy-makers to intervene early and effectively. A future study could explore the impact of other variables on the diet diversity of T2DM patients and validate DDS using biomarkers.

## Figures and Tables

**Table 1:** Characteristics of the patients included in the study.

Variables	All Patients (*n* = 390)
**Sex**	
*Male*	111 (28.5)
*Female*	279 (71.5)
**Age** (years)	57.1 ± 10.4
*<60 years*	231 (59.2)
*≥60 years*	159 (40.8)
**Smoking** (yes)	11 (2.8)
**Family history of diabetes** (yes)	280 (71.8)
**Diabetes duration** (years)	8.8 ± 7.3
*<10 years*	238 (61.0)
*≥10 years*	152 (39.0)
**Antidiabetic treatment strategies**	
*Dietary treatment alone*	11 (2.8)
*Oral anti-diabetic drugs alone, any*	237 (60.8)
*Insulin alone*	47 (12.1)
*Oral anti-diabetic drugs plus insulin*	95 (24.4)
**HbA1c (%)**	7.5 ± 1.7
*<7%*	168 (43.1)
*≥7%*	222 (59.9)
**Total number of diabetes complications/comorbidities**	
*0*	152 (39.0)
*1–2*	212 (54.4)
*≥3*	26 (6.7)
**Diabetes complications and comorbidities among participants**	
*Cardiovascular disease*	57 (14.6)
*Retinopathy*	52 (13.3)
*Neuropathy*	11 (2.8)
*Nephropathy*	25 (6.4)
*Hypertension*	184 (47.2)
**BMI** (kg/m^2^)	29.4 ± 5.0
*Normal body weight*	79 (20.3)
*Overweight*	150 (38.5)
*Obese*	161 (41.3)
**Waist circumference** (cm)	103.8 ± 12.5
*Normal*	25 (6.4)
*At risk*	365 (93.5)
**Hip circumference** (cm)	109.3 ± 12.7
**Waist-to-hip ratio**	0.9 ± 0.09
*Normal*	44 (11.3)
*At risk*	346 (88.7)
**Body fat** (%)	40.0 ± 8.8
**Fat mass index** (kg/m^2^)	12.1 ± 4.5
**Physical activity level**	
*Low*	238 (61.0)
*Moderate*	136 (34.9)
*High*	16 (4.1)

Results are expressed as *n* (%) or mean ± SEM.

**Table 2:** Relationship between diabesity and demographic, clinical, anthropometric and physical activity variables.

Independents Variables	Non-Obese (*n* = 229)	Obese (*n* = 161)	OR (95% CI)	*p*
**Sex**				
*Male*	87 (38.0)	24 (14.9)	1	
*Female*	142 (62.0)	137 (85.1)	3.497 (2.102–5.820)	<0.0001
**Age** (years)	57.9 ± 9.8	56.0 ± 11.0	0.982 (0.963–1.001)	0.070
*<60 years*	128 (55.9)	103 (64.0)	1	
*≥60 years*	101 (44.1)	58 (36.0)	0.714 (0.472–1.080)	0.110
**Smoking**				
*No*	-	-	1	
*Yes*	9 (3.9)	2 (1.2)	0.307 (0.066–1.442)	0.135
**Family history of diabetes**				
*No*	-	-	1	
*Yes*	166 (72.5)	114 (70.8)	0.921 (0.589–1.439)	0.716
**Diabetes duration** (years)	8.8 ± 6.6	8.9 ± 8.1	1.001 (0.974–1.029)	0.949
*<10 years*	138 (60.3)	100 (62.1)	1	
*≥10 years*	91 (39.7)	61 (37.9)	0.925 (0.611–1.400)	0.712
**Antidiabetic treatment strategies**				
*Dietary treatment alone*	5 (2.2)	6 (3.7)	1	
*Oral anti-diabetic drugs alone, any*	144 (62.9)	93 (57.8)	0.538 (0.160–1.814)	0.318
*Insulin alone*	30 (13.1)	17 (10.6)	0.472 (0.125–1.781)	0.268
*Oral anti-diabetic drugs plus insulin*	50 (21.8)	45 (28.0)	0.750 (0.214–2.626)	0.653
**HbA1c** (%)	7.5 ± 1.8	7.5 ± 1.6	0.991 (0.885–1.110)	0.876
*<7%*	103 (45.0)	65 (10.4)	1	
*≥7%*	126 (55.0)	96 (59.6)	1.207 (0.802–1.817)	0.366
**Total number of diabetes complications/comorbidities**				
*0*	94 (41.0)	58 (36.0)	1	
*1–2*	121 (52.8)	91 (56.5)	1.219 (0.796–1.865)	0.362
*≥3*	14 (6.1)	12 (7.5)	1.389 (0.601–3.210)	0.442
**Diabetes complications and comorbidities among participants**				
*Cardiovascular disease*				
No	-	-	1	
Yes	33 (14.4)	24 (14.9)	1.040 (0.589–1.839)	0.891
*Retinopathy*				
No	-	-	1	
Yes	34 (14.8)	18 (11.2)	0.722 (0.392–1.330)	0.296
*Neuropathy*				
No	-	-	1	
Yes	5 (2.2)	6 (3.7)	1.734 (0.520–5.783)	0.370
*Nephropathy*				
No	-	-	1	
Yes	15 (6.6)	10 (6.2)	0.945 (0.413–2.160)	0.893
*Hypertension*				
No	-	-	1	
Yes	97 (42.4)	87 (54.0)	1.600 (1.066–2.401)	0.023
**BMI** (kg/m^2^)	26.0 ± 2.5	34.3 ± 3.7	-	
*Normal body weight*	79 (34.5)	0 (0.0)	-	
*Overweight*	150 (65.5)	0 (0.0)	-	
*Obese*	0 (0.0)	161 (100)	-	
**Waist circumference** (cm)	97.8 ± 10.4	112.3 ± 10.1	1.155 (1.121–1.191)	<0.0001
*Normal*	82 (35.8)	3 (1.9)	1	
*At risk*	147 (64.2)	158 (98.1)	29.379 (9.084–95.017)	<0.0001
**Hip circumference** (cm)	103.6 ± 11.1	117.3 ± 10.2	1.160 (1.123–1.198)	<0.0001
**Waist-to-hip ratio**	0.9 ± 0.09	0.9 ± 0.08	3.732 (0.418–33.329)	0.238
*Normal*	29 (12.7)	15 (9.3)	1	
*At risk*	200 (87.3)	146 (90.7)	1.411 (0.730–2.727)	0.305
**Body fat** (%)	35.1 ± 6.7	47.0 ± 6.3	1.379 (1.289–1.475)	<0.0001
**Fat mass index** (kg/m^2^)	9.2 ± 2.3	16.2 ± 3.7	3.674 (2.694–5.010)	<0.0001
**Physical activity level**				
*Low*	115 (50.2)	123 (76.4)	1	
*Moderate*	102 (44.5)	34 (21.1)	0.312 (0.196–0.496)	<0.0001
*High*	12 (5.2)	4 (2.5)	0.312 (0.098–0.994)	0.049

Results are expressed as *n* (%), mean ± SEM or odds ratio (OR) with a 95% confidence interval (95% CI).

**Table 3:** Proportion of patients consuming each food group and average grams consumed per day of each food group, overall and by obesity status.

	All Patients (*n* = 390)	Non-Obese (*n* = 229)	Obese (*n* = 161)	*p*
**Proportion consuming**				
*Meat group*	272 (69.7)	152 (66.4)	120 (74.5)	0.084
*Fruit group*	362 (92.8)	212 (92.6)	150 (93.2)	0.824
*Vegetable group*	377 (96.7)	219 (95.6)	158 (98.1)	0.175
*Dairy group*	350 (89.7)	200 (87.3)	150 (93.2)	0.062
*Grain group*	381 (97.7)	226 (98.7)	155 (96.3)	0.118
**Average grams consumed**				
*Meat group*	87.6 ± 82.7	80.2 ± 78.7	98.1 ± 87.1	0.049
*Fruit group*	140.1 ± 94.2	144.8 ± 100.2	133.3 ± 84.7	0.508
*Vegetable group*	253.4 ± 168.4	260.9 ± 180.3	242.7 ± 149.9	0.612
*Dairy group*	207.6 ± 141.3	197.6 ± 139.9	221.9 ± 142.6	0.154
*Grain group*	290.5 ± 140.4	287.7 ± 143.2	294.3 ± 136.6	0.588

Results are expressed as *n* (%) or mean ± SEM.

**Table 4:** Energy and macronutrient intake of the study patients, overall and by obesity status.

	All Patients (*n* = 390)	Non-Obese (*n* = 229)	Obese (*n* = 161)	*p*
Energy intake (EI) (Kcal/d)	1411.4 ± 378.9	1401.3 ± 399.6	1425.8 ± 348.0	0.318
Carbohydrate (g/d)	209.6 ± 53.2	210.6 ± 57.9	208.1 ± 45.8	0.940
Carbohydrate (%EI)	60.6 ± 9.9	61.3 ± 10.2	59.5 ± 9.3	0.075
%EI from carbohydrate (45–60)	169 (43.3)	89 (38.9)	80 (49.7)	0.034
Protein (g/d)	43.5 ± 22.6	41.2 ± 22.8	46.6 ± 22.2	0.023
Protein (%EI)	12.3 ± 5.6	11.5 ± 5.5	13.3 ± 5.6	0.006
%EI from protein (10–20)	212 (54.4)	122 (53.3)	90 (55.9)	0.608
Fat (g/d)	44.3 ± 25.7	43.7 ± 26.3	45.1 ± 25.0	0.347
Fat (%EI)	27.1 ± 10.2	27.0 ± 10.4	27.2 ± 9.9	0.882
%EI from fat (<35)	301 (77.2)	176 (76.9)	125 (77.6)	0.856

Results are expressed as *n* (%) or mean ± SEM.

**Table 5:** Crude and multivariable-adjusted OR and 95% CI for the association between DDS and food groups intake with obesity.

	Crude Model	*p*	Model 1	*p*	Model 2	*p*	Model 3	*p*
**DDS**								
*Non-obese*	1		1		1		1	
*Obese*	1.335 (0.997–1.786)	0.052	1.376 (1.020–1.855)	0.037	1.458 (1.073–1.981)	0.016	1.426 (1.029–1.974)	0.033
**Meat group**								
*Non-obese*	1		1		1		1	
*Obese*	1.003 (1.000–1.005)	0.036	1.003 (1.001–1.006)	0.017	1.003 (1.001–1.006)	0.017	1.003 (1.000–1.006)	0.032
**Fruit group**								
*Non-obese*	1		1		1		1	
*Obese*	0.999 (0.996–1.001)	0.238	0.999 (0.997–1.001)	0.439	0.999 (0.997–1.002)	0.543	0.999 (0.996–1.001)	0.382
**Vegetable group**								
*Non-obese*	1		1		1		1	
*Obese*	0.999 (0.998–1.001)	0.293	1.000 (0.998–1.001)	0.536	1.000 (0.998–1.001)	0.649	1.000 (0.998–1.001)	0.473
**Dairy group**								
*Non-obese*	1		1		1		1	
*Obese*	1.001 (1.000–1.003)	0.097	1.001 (0.999–1.002)	0.281	1.001 (0.999–1.002)	0.379	1.000 (0.999–1.002)	0.627
**Grain group**								
*Non-obese*	1		1		1		1	
*Obese*	1.000 (0.999–1.002)	0.649	1.001 (0.999–1.003)	0.213	1.001 (1.000–1.003)	0.125	1.000 (0.999–1.003)	0.289

Results are expressed as odds ratio (OR) with a 95% confidence interval (95%CI). Model 1: Adjusted for age and sex. Model 2: Adjusted for age, sex, smoking and physical activity. Model 3: Adjusted for age, sex, smoking, physical activity and energy intake. DDS: Dietary diversity score.

## Data Availability

The data presented in this study are available on request from the corresponding author.

## Supplementary Materials

The following are available online at https://www.mdpi.com/article/10.3390/healthcare9091229/s1, Table S1: Association between DDS and both energy intake and consumption of all three macronutrients, Figure S1: Proportion of patients with insufficient energy intake.
