# Supplementary material for: Dietary Diversity as a Risk Factor for Obesity in Algerian Patients with Type 2 Diabetes Mellitus
Source: Healthcare (Basel). 2021 Sep 17;9(9):1229. doi: 10.3390/healthcare9091229 (PMC8468535; doi:10.3390/healthcare9091229)
Supplement: Supplementary file 1 [file healthcare-09-01229-s001.zip › healthcare-1378685-supplementary.pdf]

Supplementary Material

CONTENTS

Supplementary Table S1 Association between DDS and both energy intake and consumption of all three macronutrients.

Supplementary Figure S1 Proportion of patients with insufficient energy intake.

Supplementary Table S1 Association between DDS and both energy intake and consumption of all three macronutrients.

**Variable**	**η2**
Energy intake	0.115**
Carbohydrate	0.064**
Protein	0.329***
Fat	0.026*

Effect sizes were analyzed by Eta-squared (η^2) to study the association between DDS and both energy intake and consumption of all three macronutrients. The results were considered as *small**, *medium*** or *large**** effect sizes if $0.01 \leq \eta^2 < 0.06$, $0.06 \leq \eta^2 < 0.14$ and $\eta^2 \geq 0.14$, respectively (Cohen, 1988).

References:

Cohen, J. (1988). *Statistical power analysis for the behavioral sciences*. Hillsdale, NJ: Erlbaum.

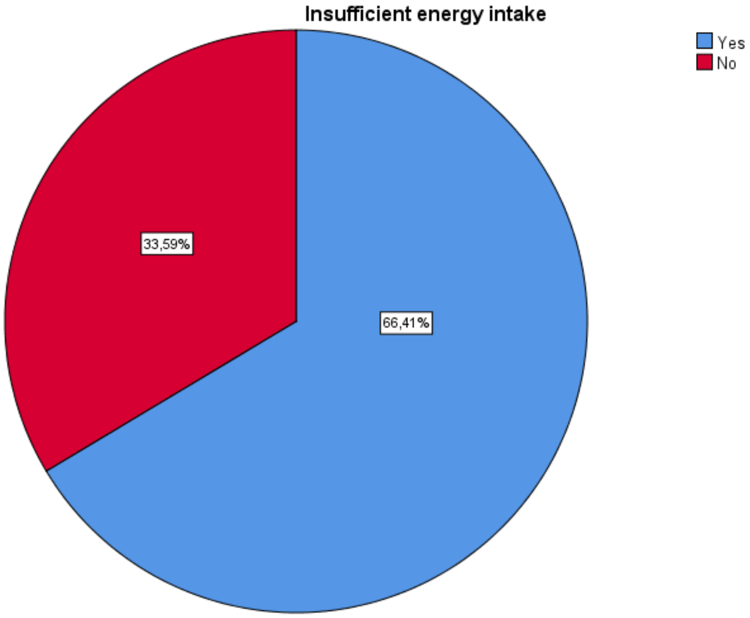

Supplementary Figure S1 Proportion of patients with insufficient energy intake. Insufficient energy intakes were found in 66.41% based on < 75% estimated average requirement.
